# Key benthic species are affected by predicted warming in winter but show resistance to ocean acidification

**DOI:** 10.1002/ece3.70308

**Published:** 2024-09-18

**Authors:** Katrin S. H. Schertenleib, Tallulah Davey, David Taylor, Nessa E. O'Connor

**Affiliations:** ^1^ Discipline of Zoology School of Natural Sciences, Trinity College Dublin Dublin 2 Ireland; ^2^ Department of Mechanical, Manufacturing and Biomedical Engineering School of Engineering, Trinity College Dublin Dublin 2 Ireland

**Keywords:** climate change, mesocosms, multiple stressors, *Mytilus edulis*, *Saccharina latissima*

## Abstract

The effects of climate change on coastal biodiversity are a major concern because altered community compositions may change associated rates of ecosystem functioning and services. Whilst responses of single species or taxa have been studied extensively, it remains challenging to estimate responses to climate change across different levels of biological organisation. Studies that consider the effects of moderate realistic near‐future levels of ocean warming and acidification are needed to identify and quantify the gradual responses of species to change. Also, studies including different levels of biological complexity may reveal opportunities for amelioration or facilitation under changing environmental conditions. To test experimentally for independent and combined effects of predicted near‐future warming and acidification on key benthic species, we manipulated three levels of temperature (winter ambient, +0.8 and +2°C) and two levels of pco_2_ (ambient at 450 ppm and elevated at 645 ppm) and quantified their effects on mussels and algae growing separately and together (to also test for inter‐specific interactions). Warming increased mussel clearance and mortality rates simultaneously, which meant that total biomass peaked at +0.8°C. Surprisingly, however, no effects of elevated pco_2_ were identified on mussels or algae. Moreover, when kept together, mussels and algae had mutually positive effects on each other's performance (i.e. mussel survival and condition index, mussel and algal biomass and proxies for algal productivity including relative maximum electron transport rate [rETRmax], saturating light intensity [*I*_k_] and maximum quantum yield [*F*_v_/*F*_m_]), independent of warming and acidification. Our results show that even moderate warming affected the functioning of key benthic species, and we identified a level of resistance to predicted ocean acidification. Importantly, we show that the presence of a second functional group enhanced the functioning of both groups (mussels and algae), independent of changing environmental conditions, which highlights the ecological and potential economic benefits of conserving biodiversity in marine ecosystems.

## Graphical abstract

We identified the effects of ocean warming in winter on mussels and algae and found evidence of resistance to acidification. We also discovered a mutually facilitative interaction between mussels and algae that was robust to changing environmental conditions. The importance of conserving intact communities to maintain associated rates of ecosystem functioning is shown.

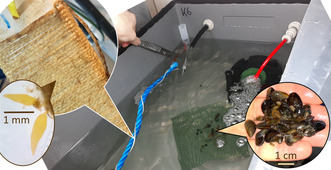

## 1 INTRODUCTION

The fate of key coastal species is a major concern in changing climate conditions (Cooley et al., 2022; IPCC, 2022; Pörtner et al., 2021). Climate change adds to other anthropogenic stressors, such as habitat modifications, exploitation or pollution, and can intensify their effects on the distribution, functioning and production of marine species (Bowler et al., 2020; Gissi et al., 2021; O'Hara et al., 2021). Coastal regions are particularly exposed to the cumulative effects of multiple stressors, which puts the ecosystem services they provide at risk, for example, their potential to provide food, regulate coastal erosion, recycle nutrients, sequester carbon, support recreational activities and sustain cultural identity (Bowler et al., 2020; Cooley et al., 2022). Predicting the cumulative impacts of multiple stressors on key coastal species, including how species interactions can be modified by climate change, and how species interactions can alter species' sensitivity to certain stressors, is crucial for managing and protecting vulnerable ecosystem services (Brooks & Crowe, 2018; Cooley et al., 2022; Gissi et al., 2021; Kéfi et al., 2024; Keyes et al., 2021; Turschwell et al., 2022).

Climate change and its effects on marine benthic species have been studied extensively, yet impacts are highly variable across taxonomic groups and up‐scaling results to communities or ecosystems remains challenging because of the interacting effects of multiple stressors and the indirect effects of biotic interactions (Doney et al., 2020; Gissi et al., 2021; Hoppit & Schmidt, 2022; Turschwell et al., 2022). Warming directly affects metabolic rates and physiological processes along thermal performance curves (Dell et al., 2011; Pörtner, 2010; Roma et al., 2021). Typically, performance first increases with warming, as metabolic costs are more efficiently met by the rate of the supplied energy until it culminates at an optimum temperature, beyond which costs outbalance any achievable supply and performance declines rapidly (Clarke & Gaston, 2006; Gillooly et al., 2001; Lemoine & Burkepile, 2012; Pörtner, 2010). Whether localised warming enhances or impairs performance is determined by the temperature range on the thermal performance curve to which an organism's phenology is acclimatised in its habitat (Vasseur et al., 2014). Seasonal environments may determine where organisms are on their performance curves and seasonal adaptations may influence the shape, or temperature range, of an organism's performance curve (Huey et al., 2012). Species have evolved various strategies to adapt to winter conditions, for example, with reduced activity, but we have a limited understanding of how species cope with changing winters or how this affects their trophic and non‐trophic interactions (Dinh et al., 2023). The biological mechanisms underlying the effects of ocean acidification are not understood fully, however, we know that changes in water carbon chemistry can affect fundamental processes, such as photosynthesis in algae or calcification in bivalves (Doney et al., 2020 and references therein; Doo, Kealoha, et al., 2020; Lutier et al., 2022). Additionally, multiple climate change‐related stressors may interact, or modify the sensitivity of an organism to certain stressors (Bruder et al., 2019; Cahill et al., 2013; Melzner et al., 2013; Turschwell et al., 2022). Altered species interactions may affect ecological networks indirectly as strongly as direct stressor effects on species performances, thus, we need to identify consistent generalities to support management and conservation (Bruder et al., 2019; Cahill et al., 2013; Doney et al., 2020; Gaylord et al., 2015; Turschwell et al., 2022).

Bivalves and macroalgae are important habitat‐forming components of coastal marine ecosystems (O'Connor & Crowe, 2007; Wear et al., 2023), where altered community compositions may profoundly change ecosystem functioning and provisioning (Cooley et al., 2022; Hoppit & Schmidt, 2022; Lemasson et al., 2017). Calcifying species, such as bivalves, tend to respond to ocean warming and acidification with increased metabolic costs, reduced growth, reproduction and calcification (Doo, Kealoha, et al., 2020; Hoppit & Schmidt, 2022; Lemasson et al., 2018; Lutier et al., 2022; Sadler et al., 2018). Fleshy algae, on the other hand, tend to show resistance and may even benefit from warmer and acidified conditions because of increased CO_2_ available for photosynthesis at accelerated metabolic rates (Hoppit & Schmidt, 2022; Mooney‐McAuley et al., 2016; Stewart et al., 2013). Other ecosystems dominated by fleshy macroalgae, however, suffer from extreme heat events (Smale et al., 2019), increased grazing, competition or impaired reproduction (Veenhof et al., 2022), which may alter community compositions and even cause shifts from complex macroalgal habitats to degraded algal turf communities (Provost et al., 2017). Understanding the effects of warming and acidification on these key species, including interactions with other species closely associated with these biogenic habitats, is therefore a research priority (Hobday et al., 2016; Tagliarolo et al., 2018; Turschwell et al., 2022; Wernberg et al., 2012).

Marine macrophytes, such as macroalgae and seagrass, have the potential to mitigate negative acidification effects on calcifiers, such as bivalves, by removing CO_2_ from the water and increasing pH, thus, acting as a local buffer to acidification (Doo, Leplastrier, et al., 2020; Jiang et al., 2022; Jiang & Fang, 2021; Young, Sylvers, et al., 2022). In addition to increasing mean pH with increasing macrophyte biomass, diurnal pH fluctuations become more pronounced, which offers bivalves (e.g. mussels and oysters) temporal refuge from acidification stress that can be used for increased calcification activity (Edworthy et al., 2023; Ricart et al., 2021; Wahl et al., 2018). Integrated multi‐trophic aquaculture was first established to utilise surplus nutrients produced by cultivated finfish through co‐cultivation with detritivores, filter‐feeding bivalves and macroalgae (Mooney‐McAuley et al., 2016). More recently, the commercial potential of co‐culturing bivalves and macroalgae to enhance shellfish production has been recognised (Hamilton et al., 2022). Macroalgal aquaculture is a rapidly growing industry with demands for bioenergy, food, pharmaceutical and fertiliser industries, producing ca. 37 million tonnes of algal biomass annually (FAO, 2024; Mooney‐McAuley et al., 2016). Whilst approximately 19 million tonnes of marine molluscs, mainly bivalves, are produced per year, which represents just over half of coastal and marine animal aquaculture (FAO, 2024). The impact of climate change on aquaculture production is largely unknown and predictions of ecological and economic impacts vary tremendously (Forbes et al., 2022; Gubbins et al., 2013; Hengjie et al., 2023; Theuerkauf et al., 2022; Troell et al., 2022). Therefore, understanding the mechanisms driving the combined effects of warming and acidification on co‐cultures of bivalves and macroalgae, including adaptation and mitigation of a changing climate, is of ecological and commercial interest (Duarte et al., 2017; Jiang & Fang, 2021; Young, Sylvers, et al., 2022).

Much research has been conducted on single species where the effects of ocean warming and acidification were based on or exceeding the worst‐case scenarios of IPCC climate change predictions (Armstrong et al., 2022; Geraldi et al., 2020; Knights et al., 2020; Navarro et al., 2016; Zhang et al., 2022). These studies help understand population dynamics and community structures under extremely predicted future conditions (Lemoine & Burkepile, 2012; Rall et al., 2010). Incorporating more moderate near‐future experimental treatments, however, is required to identify how species and ecosystems may respond within the next few decades or in moderate climate change scenarios by 2100, and to identify their potential for adaptation (Geraldi et al., 2020).

**FIGURE 1:**
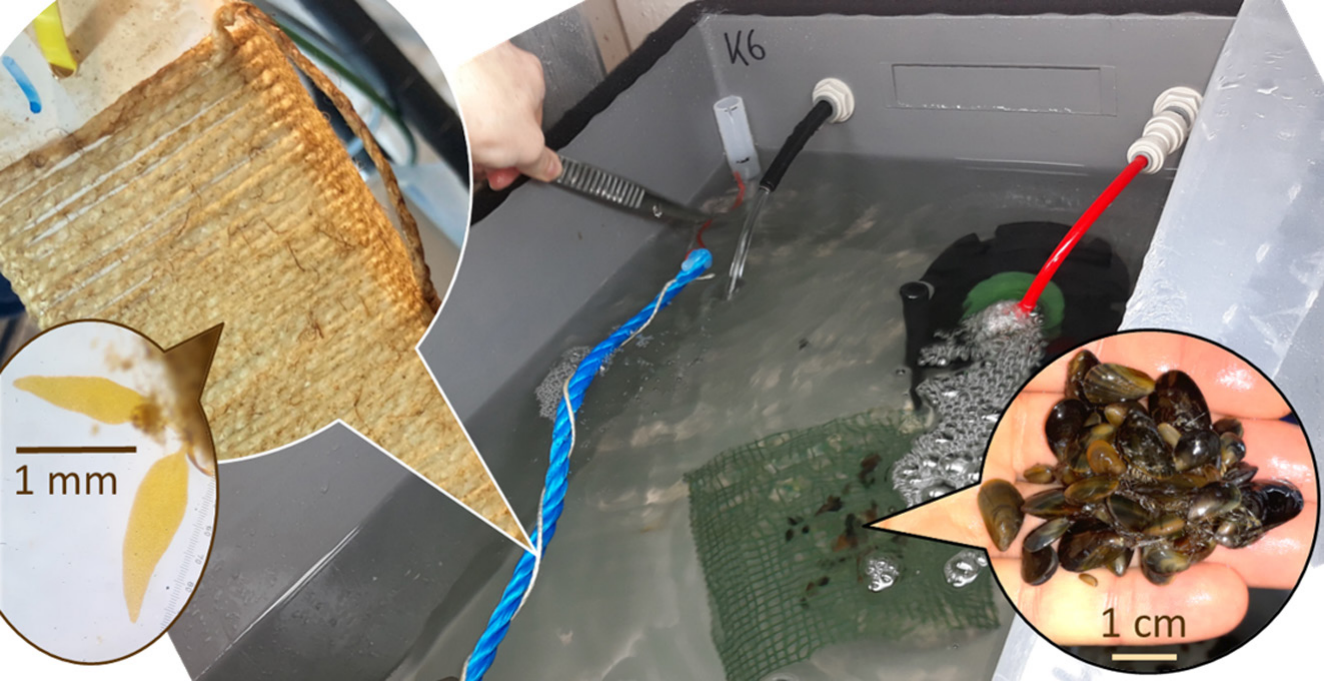
Sugar kelp sporophytes (*Saccharina latissima*) on the seeded string (left) and blue mussel spat (*Mytilus edulis*) placed on mesh tiles (right) were grown in separate mesocosms or together at different levels of winter warming and/or simulated acidification.

In the current study, we tested empirically for the effects of increased winter temperature and/or ocean acidification on blue mussels (*Mytilus edulis*) and sugar kelp sporophytes (*Saccharina latissima*) grown together and separately with a fully factorial experimental design (Figure 1). Temperature (winter ambient [8–13°C]; +0.8°C; +2°C) and pco_2_ (ambient, 450 ppm; elevated, 645 ppm) were manipulated based on predicted sea surface temperature and atmospheric pco_2_ of the Irish Sea under a moderate climate change scenario by the year 2100, or sooner in the case of a more extreme scenario (IPCC, 2013, 2014; Jacob et al., 2014). We quantified the effects of these abiotic experimental treatments (temperature and pco_2_) on several proxies for the functioning of mussels and algae including mussel mortality, mussel biomass, mussel condition index, clearance rates, mussel shell and byssus strength, algal biomass and photosynthetic performance (i.e. relative maximum electron transport rate [rETRmax], saturating light intensity [*I*_k_], light harvesting efficiency [*α*] and maximum quantum yield [*F*_v_/*F*_m_]). Additionally, we tested whether the functioning of mussels and algae differed in the presence or absence of each other. We hypothesised that both species would respond to warming with increases in all processes quantified, except mussel mortality, which we expected to decrease, because the experiment took place in winter, that is, at the lower end of the temperature range seasonally encountered by our study organisms. Simultaneously, we expected acidification to have a negative effect on mussels and a positive effect on algal production. Additionally, we hypothesised that the presence of the algae would strengthen the positive effects of warming, and act as a local buffer to mitigate the negative effects of acidification on mussels, whilst the presence of mussels would enhance the productivity and photosynthetic performance of the algae. As a consequence, we expected the total accumulated biomass of mussels and algae to be greater in the treatments where they were kept together compared to the sums of biomass of treatments containing just mussels or algae.

## 2 METHODS

### 2.1 Experimental design

**FIGURE 2:**
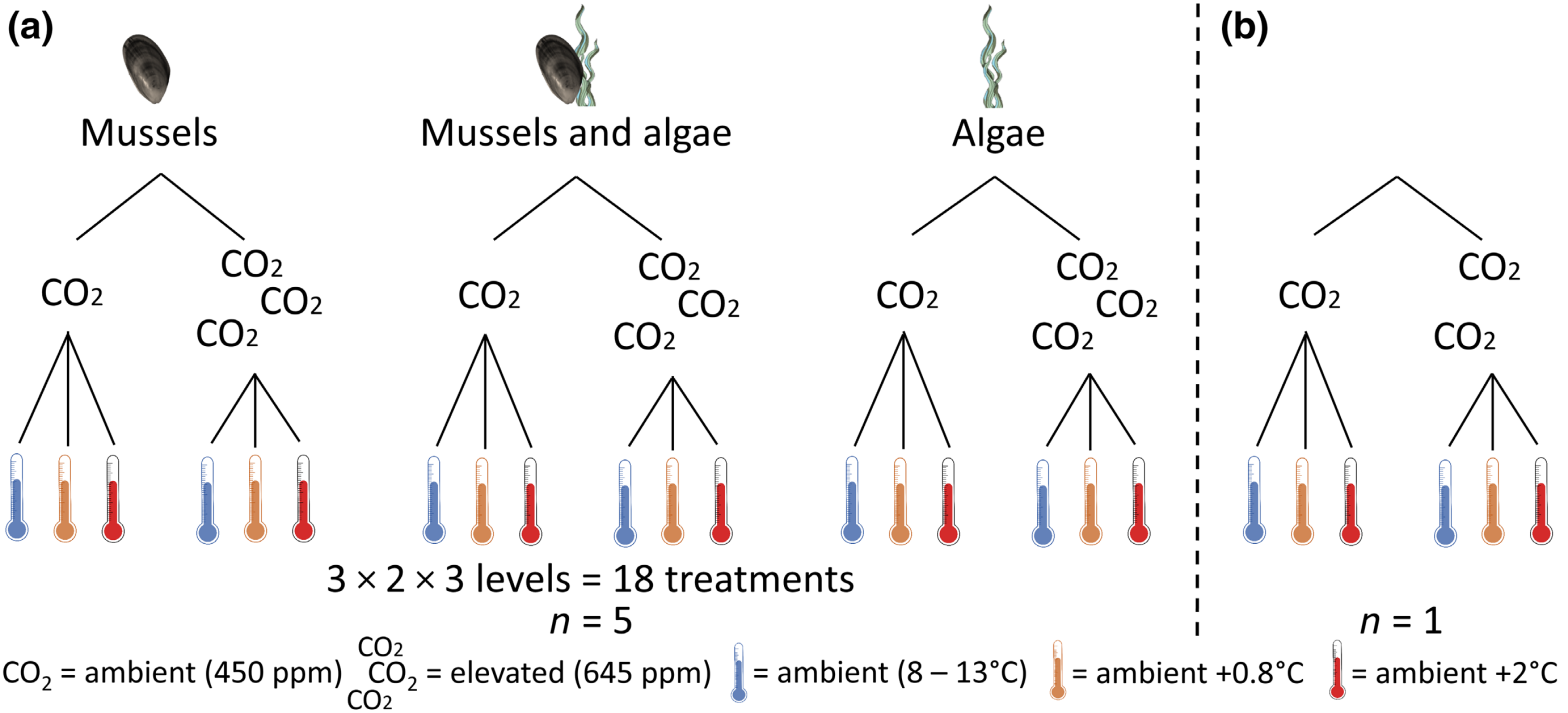
Experimental design testing for effects of winter warming (three levels) and acidification (two levels) on mussels, and on algae, separately and together, to also test for interactive effects of mussels and algae on each other (a) and to monitor the abiotic treatment manipulation in the absence of biota (b).

The experiment simultaneously tested the single and combined effects of the abiotic factors warming and acidification on mussels or algae separately, in addition to testing for the effects of mussels and algae on each other (Figure 2a). Specifically, we tested whether the presence of a second functional group (mussels or algae) would alter the effects of the abiotic factors on either group. Water temperature was manipulated at three levels: winter ambient, +0.8°C, and +2°C. pco_2_ was manipulated at two levels to simulate acidification with the corresponding pH: ambient (450 ppm) and elevated (645 ppm). In total, 18 treatments (*n* = 5) were distributed randomly across 90 mesocosms and the factorial design enabled the testing of the independent and combined effects of all factors and their respective levels (Figure 2a). The abiotic treatments simulated predicted conditions in Ireland based on a moderate climate change scenario in the year 2100 (i.e. +0.8°C and 645 ppm) and even warmer conditions (+2°C by the year 2100), which may prevail before the year 2100 approaching an extreme business‐as‐usual scenario (IPCC, 2014, 2019; Jacob et al., 2014). Six additional mesocosms that contained only seawater but no biota (that can influence pH [Lowe et al., 2019]) were established to monitor variation in the abiotic conditions at each combination of temperature and pco_2_ levels (Figure 2b). Mussels and algae were collected locally and acclimatised to laboratory conditions at ambient temperature and pco_2_ for 1 week before temperature and pco_2_ manipulations started (Kong et al., 2019). Response variables were taken after 6–7 weeks at manipulated abiotic conditions, which were stopped after 45 days: mussel clearance rates were measured on days 35–36; mussel mortality, biomass and condition index were determined on day 41; mussels for shell strength tests were collected on day 41, prepared for testing 5 days later and tests conducted after 5 months; mussels were cut off byssus stems on day 41 and byssus strength tests conducted 4 days later. Algal photosynthetic performance was assessed on day 39; biomass was measured on day 42 and kelp abundance was determined on days 43 and 44.

### 2.2 Experimental set‐up

The experiment was carried out using the experimental mesocosm platform QIMS (Quantifying the Impacts of Multiple Stressors; described in detail in Schertenleib et al., 2024) at Trinity College Dublin, Ireland, from 28/10/2020 to 18/12/2020. The set‐up comprised of 96 independent mesocosms that were opaque polypropylene boxes (40 × 60 × 28 cm) filled with 32 L of seawater. The seawater was collected from a 20 m depth at the Irish continental shelf (supplied by Seahorse Aquariums) and had a salinity of 34.3. In each mesocosm, the water was continuously recirculated at 130 L/h using small aquarium pumps (OptiMax 500, Oase Living Water), which ensured homogenous mixing. The seawater flow returned into each mesocosm at an angle opposite the pumps to maximise mixing and increase surface gas exchange. Mesocosms were aerated at 15.5 mL/min via aquarium sponge filters (PK200 for <200 L, Xinyou Aquarium) that ensured nutrient cycling by providing a settlement surface for microorganisms, such as denitrifying bacteria. Lids of transparent polypropylene (Foliarex UV4 Greenhouse Film) were attached to the mesocosms and captured the individual, ambient or pco_2_‐enriched atmospheres in 16 L headspaces above the seawater. Illumination of 50 ± 4 μmol/m^2^s (mean ± SD; penetrating through the lids) was provided by LED lights with standard artificial daylight colour (Radium RaLED DAMPPROOF; 6500 Kelvin), set to a 10:14 h light:dark cycle, reflecting average Irish autumn daylight conditions. Oxygen levels were measured weekly and remained saturated >8.2 mg/L. Halfway through the experiment, the water was drained and replaced with fresh seawater to maintain high water quality. During this process, mussel faeces and biofilm were removed from the mesocosm bottoms. Mussels and algae, which can both occur in the intertidal in the wild, were disturbed as little as possible and exposure to air was minimised to 60 min.

### 2.3 Temperature manipulation

Mesocosm water temperature was manipulated using a separate, facility‐wide cooling water circuit with individual heat exchangers at each mesocosm. When recirculating the mesocosm water, the recirculation tubes passed in opposite current direction through cooling water tubes. Three different temperature levels were set with valves leading the recirculation tubes through different lengths of heat exchangers. Three parallel‐connected beer chillers (MF Refrigeration Ltd, Midi 6 Coil R290 Hydrocarbon Cooler, 2 kilowatts capacity each) continuously cooled approximately 2000 L of fresh water, circulated through the facility at a rate of 8200 L/h.

After temperature manipulation started, target temperatures were reached in 4–6 h. Room temperature decreased owing to outside temperatures over the course of the experiment and the coldest (‘ambient’) temperature level ranged between 8.5°C ± 0.07 and 12.4°C ± 0.07 (means ± SE). The medium temperature level was warmer by 0.8°C ± 0.0 and the warmest level by 2.0°C ± 0.1 (means ± SE). Temperature was logged every 10 min in 42 mesocosms using HOBO pendants (type MX2201, Temperature/Light 64 K; two randomly deployed per treatment, one per mesocosm without biota for manipulation monitoring) and measured weekly in all mesocosms using a handheld digital thermometer (Fisherbrand Traceable Kangaroo Thermometer).

### 2.4 pco_2_ manipulation and pH

pco_2_ was manipulated through the air lines aerating each mesocosm. Ambient air (450 ppm, from Dublin City) was distributed to mesocosms using large blower pumps, whilst an alternative air line provided a CO_2_‐enriched air mixture. Valves on each mesocosm controlled which of the two air lines were supplied. To elevate pco_2_, pure CO_2_ was injected into a mixing vessel prior to distribution in the facility. The concentration was controlled using a gas analyser (LI‐COR, LI‐830) in combination with a professional aquarium computer (GHL, ProfiLux 3) and logged every second. Ambient pco_2_ was monitored every minute using a CO_2_ data logger (Driesen‐Kern, DK660).

Ambient pco_2_ was 452 ± 0.12 ppm, whilst elevated pco_2_ was 645 ± 0.04 ppm (mean ± SE). The corresponding pH in the mesocosms without biota was (mean ± SE) 8.09 ± 0.004 at ambient pco_2_ and 7.97 ± 0.003 at elevated pco_2_, which was confirmed twice weekly. The elevated pco_2_ level was reached in the air mixture within minutes of starting the treatment manipulation, whilst the pH in the seawater was adjusted in less than 18 h.

### 2.5 Study organisms

One‐year‐old mussel spat (*M. edulis*) was obtained from the seeded rope at 1 m depth in Killary Fjord (Killary Fjord Shellfish; http://killaryfjordshellfish.com/who.html) in October 2020 (in situ water temperature 11.2°C, pH 8.24) and kept moist and cool for transport until placed in 13°C aerated seawater in Trinity College Dublin. After 5 days, 3.01 g ± 0.01 (mean ± SE) of live mussel spat wet biomass, made up of 30–40 mussels with an average individual wet biomass of 0.096 mg ± 0.001 (mean ± SE) and shell lengths between 7 and 16.5 mm, were assigned randomly to mesocosms. In the mesocosms, the mussels were placed on garden mesh tiles (15 × 15 cm polypropylene with 7 mm single mesh diameter), 2 cm above the bottom and close to the recirculation return to ensure similar hydrodynamic conditions. After 2 weeks, all had attached either to the mesh or to other mussels. Empty mesh was similarly deployed in all other mesocosms.

Mussels were fed daily with a concentrated marine microalgal mix (Reed Mariculture Shellfish Diet 1800; 0.7 mL Shellfish Diet per gram live spat) to ensure starvation was not the underlying cause for any putative effects in response variables (Thomsen et al., 2013). The total amount of Shellfish Diet per mesocosm during the first week of the experiment was 2 mL, which was increased to 2.5 mL in weeks 2 and 3, and to 2.75 mL in week 4, anticipating growth of the mussels. After week 3.5 the mussels no longer cleared the water, possibly because of mortality, thus, the food dosage was reduced to 1.7 mL daily per mesocosm containing mussels until the end of the experiment.

Algal sporophytes were obtained on seeded aquaculture string from the Queen's University Marine Laboratory (QML) in Portaferry. The string had been sprayed with a *S. latissima* (kelp, brown macroalgae) gametophyte culture and was grown following standard operation procedure hatchery conditions (Gorman, 2014) for 11 weeks prior to addition to the mesocosms. Both in the mesocosms and in the hatchery, the kelp sporophytes grew slowly and at low density compared to previous trials, possibly compromised following reduced care in the hatchery during the 2020 Covid‐19 pandemic lockdowns (e.g. hatchery room lights had been kept switched off, instead of adding to the culture light and reducing light availability for the juvenile kelp). As a result, kelp individuals were microscopic when the seeded string was deployed into the mesocosms but were expected to grow rapidly.

In the treatments that included algae, 65 cm of seeded string were weighed and randomly assigned to one of the 60 respective mesocosms. To establish this experimental treatment, the seeded string was wrapped around 60 cm long polypropylene header ropes (10 mm diameter), which were fastened 1 cm below the water surface diagonally to the bottom area after soaking in seawater for 2 days. A freshly prepared solution of premixed f2 powder (Varicon Aqua, 0.5 mL f2 solution L^−1^ seawater) was added to the mesocosms with seeded string weekly because we expected the kelp to grow and deplete the nutrients over a week.

### 2.6 Data collection

#### 2.6.1 Mussel mortality

Every 2–4 days, the mesocosms were checked for dead mussels, which were identified by their shells gaping open and no closing response to physical stimulation or by lack of attachment. Mussels that died during the acclimatisation period were replaced prior to the temperature and pco_2_ manipulation, using similar‐sized live individuals that had been kept at similar lab conditions. After abiotic manipulations had started, dead mussels were removed and recorded to quantify mortality. The total amount of dead mussels per mesocosm was used for data analyses. Mortality was independent of mussel size (Supplementary Material A.1: Appendix S1), which enabled averaging and standardising mussel response variables that were measured per mesocosm (e.g. clearance rate and accumulated biomass) according to individuals.

#### 2.6.2 Mussel biomass and condition index

The mussels were retrieved on their mesh tiles from the mesocosms 51 days after deployment, that is, after 41 days at manipulated conditions, to measure total accumulated wet mussel biomass per mesocosm, which was then standardised by the number of alive mussels. Accumulated biomass per mussel was calculated as the difference between the initial and final mean individual wet biomass (mg). To calculate the condition index, a subsample of five mussels of similar shell length was selected per mesocosm and dissected into shells and flesh, which were then weighed and dried at 80°C until dry weight remained constant. Following Lucas and Beninger (1985), the condition index was calculated to assess the bivalves' physiological state, as the shell is a product of cumulative growth whilst the flesh represents recent metabolic activity that may be reduced under stress:

$$\text{Condition Index}=\frac{\mathrm{Dry}\;{\text{weight}}_{\text{Flesh}}}{\mathrm{Dry}\;{\text{weight}}_{\text{Shell}}}*100=\left[\%\right]$$

#### 2.6.3 Mussel clearance rates

Clearance rate samples were taken on the 35th and 36th day of manipulated temperature and pco_2_ conditions. On the previous day, the mussels had received a reduced feeding dosage of 1 mL shellfish diet per mesocosm. To measure clearance rates, mussels were given a 1.7 mL Shellfish Diet per mesocosm. After allowing 10 min for homogenous dispersal, the first of three 50 mL samples was drawn from each mesocosm to determine the initial cell concentration (T0). Two (T1) and 17 h (T2) after feeding, additional water samples were taken. The samples were stored at 4°C and stirred before four coulter counter cell counts were conducted from a 20 mL subsample (Beckman Coulter Counter Z Series, aperture 100 μm, Kd: 59.29, sampling volume: 0.5 mL and count of particles between 6 and 19 μm). Each first count was discarded (to ensure the coulter counter tubes were flushed and only contained the current sample) and the mean was taken from the subsequent three counts.

Clearance rates were calculated following Coughlan (1969):

$$\text{Clearance rate}=\frac{M}{n*t}*\ln\left(\frac{{\text{conc}}_{T0}}{{\text{conc}}_{T1}}\right)=\left[\frac{L}{\text{mussel}*h}\right]$$

where *M* is the volume of the suspension, *n* is the number of animals per mesocosm, conc_T0_ and conc_T1_ are the concentrations of the suspension at the start (T0) and after time *t*. Samples from the mesocosms that contained no experimental organisms, and were used for monitoring the manipulated abiotic factors, confirmed low background particle load and continuous counting accuracy throughout the measurements.

#### 2.6.4 Shell strength

To analyse shell strength, the force needed to break them was determined using a materials testing machine for compression tests (ZwickRoell zwickiline Z2.5) at the Department of Mechanical, Manufacturing & Biomedical Engineering, Trinity College Dublin. Three mussels of each mesocosm were collected on the last day of the experiment, that is, after 41 days at manipulated conditions, and stored for 5 days at 4°C before they were dissected into shell valves and flesh. Visibly intact right shell valves were placed individually into the machine with the shell valve openings lying flat on the machine in a similar orientation (Mackenzie et al., 2014). A load cell of 20 N was used for smaller shells and 200 N for the largest. Force was applied from the top at a speed of 200 mm/min until shell failure occurred. The force applied was logged at 0.01 s intervals using testXpert II software (ZwickRoell) that determined the maximum applied force (*F*_max_). Sixty shells broke visibly during dissection and were discarded, and 19 tested shells were excluded from analysis because they cracked multiple times instead of showing one clear failure event. A total of 101 shell‐breaking tests were available for data analysis, with shells from all but five (each from different treatments) of the 60 mesocosms and seven to 11 shells per treatment. Shell length (Supplementary Material A.1: Appendix S1) was used to standardise *F*_max_ and the average applied force per mm (N/mm) was calculated for each mesocosm and used for data analysis.

#### 2.6.5 Byssus strength

After recording wet biomass, mussels were cut off the mesh tiles at the byssus stem, leaving the byssus threads as intact as possible. Areas where the byssus threads clearly belonged to one single mussel were marked and the mesh tiles were returned to their mesocosms for intermediate storage. Four days later, the mesh tiles were individually fastened to a materials testing machine for traction tests (ZwickRoell zwickiline Z2.5; load cell: 20 N; Department of Mechanical, Manufacturing & Biomedical Engineering, Trinity College Dublin) in the centre below the byssus cluster and parallel to the bottom of the machine. A plastic‐wrapped wire was fed centrally through the cluster so that the cluster stem was positioned in a bend of the wire, with approximately equal amounts of byssus threads on each side (exact numbers could not be determined; we estimated a range of 15–100 per individual). The wire ends were clamped into the top machine end, which pulled the wire vertically away from the mesh at 5 mm/min (Bouhlel et al., 2017), exerting traction on the byssus cluster until all the byssus threads on one side of the wire ruptured. All tests were conducted in the air. The applied force was logged at 0.1 s intervals using testXpert II software (ZwickRoell), recording the succession and magnitude of applied tensile force and load drops over time. In adult *Mytilus californianus*, large drops in loads can be assigned to single threads breaking and the sum of individual load drops exceeds the maximum force applied to a whole byssus cluster (Bell & Gosline, 1996). The many and delicate byssus threads of the juvenile *M. edulis* used in this study did not allow us to link load drops to individual observed thread ruptures. Analyses of all load drops that were recorded showed that load drops <0.01 N occurred more than five times as often as the next force range. This indicates the background noise of the tests and justifies the load drop limits considered for analysis as >0.01 N. The average load drop >0.01 N was used as a proxy for byssus strength. A total of 43 tests were conducted on byssus clusters from 34 mesh tiles. Test results from the same mesh tile were averaged and data of three random mesocosms per treatment were used for analysis, except for the two acidified mussel‐only treatments at increased temperatures, for which only two mesh tiles were available for byssus strength testing.

#### 2.6.6 Algal biomass

The kelp sporophytes on the seeded strings grew unexpectedly slowly, presumably because of unusual hatchery conditions due to pandemic lockdowns (described previously). Over time we observed that microphytobenthos (primarily benthic diatoms and, to a lesser extent, green algae) grew on the seeded string, resulting in a microscopic, mixed algal assemblage on the seeded strings. Instead of quantifying the biomass of individuals of kelp at the end of the experiment as planned, the total accumulated biomass of algae associated with seeded string was quantified. The final abundance of kelp individuals, which had reached up to 1.4 mm in length at the end of the experiment, was counted using a dissection microscope.

#### 2.6.7 Photosynthetic performance

The photosynthetic performance of the algae present in the mesocosms was tested using a pulse amplitude modulation (PAM) fluorometer (DIVING‐PAM‐II, Walz) on the 39th day at manipulated abiotic conditions. A leaf clip (DIVING‐LC, Walz GmbH) was connected to the seeded string to eliminate light and ensure consistent spacing of the optic fibre with the algae. Rapid light curves (RLCs) were taken on ambient‐light acclimated algae to assess differences in potential photosynthetic performance. Prior to starting the RLCs, tissue was quasi‐dark adapted for a few seconds to allow re‐oxidisation of the primary electron acceptor (Randall et al., 2019; Schreiber, 2004). Relative electron transport rates were determined at steps of increasing actinic light intensity, from which the DIVING‐PAM‐II calculated the relative maximum electron transport rate, that is, photosynthetic capacity rETRmax, as well as the saturating light intensity *I*_k_, and the initial slope of the RLC, that is, the light‐harvesting efficiency *α* (Randall et al., 2019). After 15 min of dark acclimation, a different section of the seeded string was then used to measure the maximum quantum yield *F*_v_/*F*_m_, calculated as $\frac{F_{v}}{F_{m}}=\frac{F_{m}-F_{0}\;}{F_{m}}$ where *F*_m_ represents the maximal fluorescence after a saturating light pulse and *F*_0_ is the steady‐state fluorescence under weak initial illumination before the light pulse (Miranda et al., 2019). The higher the yield, the more suitable the conditions (Bilger et al., 1995).

#### 2.6.8 Total accumulated biomass

The total accumulated biomass for mussels and algae that were kept in the same mesocosm was calculated by adding the accumulated algal biomass to the accumulated mussel biomass. To compare the total accumulated biomass of mussels and algae that had been kept together and the sum of accumulated biomass of mussels kept on their own and algae kept on their own in the same abiotic treatments, each possible combination of accumulated biomass per mesocosm of mussels kept on their own (*n* = 5) and algae kept on their own (*n* = 5) was calculated (for analysis *n* = 5*5 = 25).

### 2.7 Data analyses

To test hypotheses, three‐way analyses of variance (ANOVA) were performed using temperature (three levels), pco_2_ (two levels) and the presence of a second functional group (two levels; that is, mussels vs. mussels and algae or algae vs. mussels and algae) as fixed orthogonal factors and including all possible interactions based on 5 replicates (mesocosms) per treatment. Data of rETRmax and *I*_k_ were log‐transformed to meet the assumptions of ANOVA. The normality of errors was confirmed by plotting histograms of the residuals and applying Shapiro–Wilks tests. Homogeneity of variances was tested using Levene's tests and by plotting the residuals as a function of the fitted values. To test for autocorrelation in the residuals, Durbin–Watson tests were conducted, and the presence of influential data points was assessed using Cook's distance. Data of mussel shell strength and mussel byssus strength were slightly unbalanced, hence ANOVA type 3 sums of squares were considered. When the ANOVA indicated differences between more than two treatment levels, Tukey's Honest Significance Differences were calculated as post‐hoc tests. To assess whether the final amount of kelp sporophytes differed amongst treatments, a generalised linear model of the family quasipoisson was applied. To test for differences in total accumulated biomass when mussels and algae were kept together and the sums of accumulated mussel and algal biomass when kept separately, the sums of all possible biomass combinations of mesocosms that had only mussels and only algae were calculated for each abiotic treatment (temperature levels crossed with pco_2_ levels), which yielded 25 samples per abiotic treatment. Variances were heterogeneous between these groups and the corresponding five samples of mesocosms in which mussels and algae had been kept together at the same abiotic treatments, thus the Scheirer–Ray–Hare test was used for analysis, followed by the Dunn test for post‐hoc comparisons (Mangiafico, 2016).

All statistical analyses and data visualisation were conducted in R version 4.2.1 (R Core Team, 2022) using R Studio version 2022.07.2 (RStudio Team, 2022) and the packages tidyverse version 1.3.2 (Wickham et al., 2019), car version 3.1.1 (Fox & Weisberg, 2019), Hmisc version 4.7‐2 (Harrell Jr, 2022), rcompanion version 2.4.34 (Mangiafico, 2023) and FSA version 0.9.5 (Ogle et al., 2023). Byssus strength test files were prepared and the average load drop >0.01 N was calculated in Microsoft Excel.

## 3 RESULTS

### 3.1 Mussel responses

**FIGURE 3:**
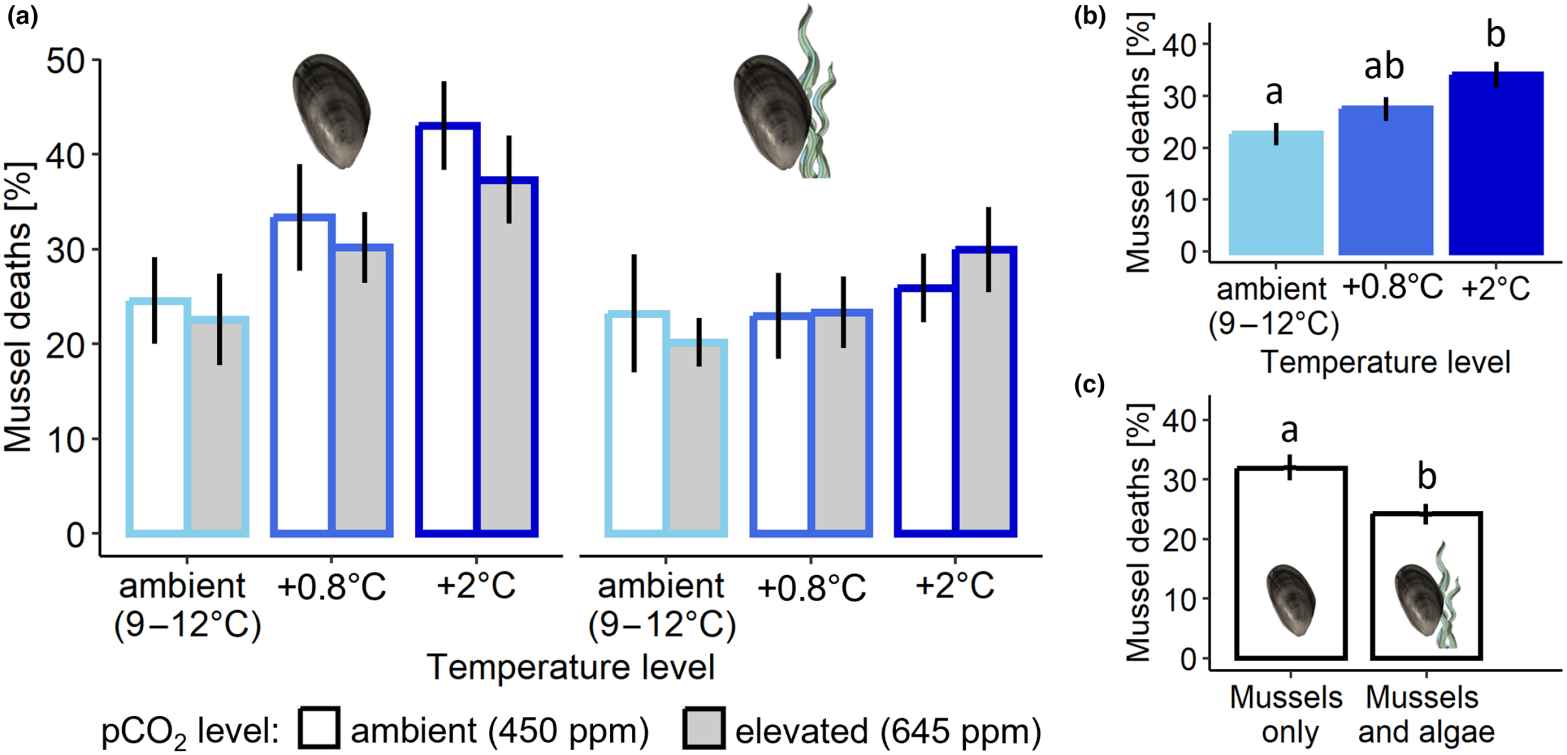
(a) Mean (±SE) of mussel deaths in all experimental treatments at winter ambient (light blue), +0.8°C (blue) and +2°C (dark blue) temperature and at ambient (white bars) and elevated (grey bars) pco_2_, in the absence (left panel) or presence (right panel) of algae, *n* = 5; (b) shows the effect of temperature on mussel deaths based on these pooled treatments and (c) shows the effect of the presence of algae on mussel deaths based on these pooled treatments without and with algae. Significant differences amongst groups of means are indicated by lowercase letters (*p* < .01).

We identified several differences amongst the temperature treatments on the mussel responses quantified and importantly no interactions were identified amongst any of the factors on any of the mussel response variables. Specifically, we identified a positive effect of temperature on mussel mortality (*F*_2,48_ = 6.382; *p* = .004) and post‐hoc tests indicated significant differences (*p* = .002) between the winter ambient and the warmest (+2°C) temperature level (Figure 3a,b). pco_2_ had no effect on mussel mortality (*F*_1,48_ = 0.37; *p* = .549). Fewer mussels died when algae were present (*F*_1,48_ = 8.403; *p* = .006; Figure 3c).

**FIGURE 4:**
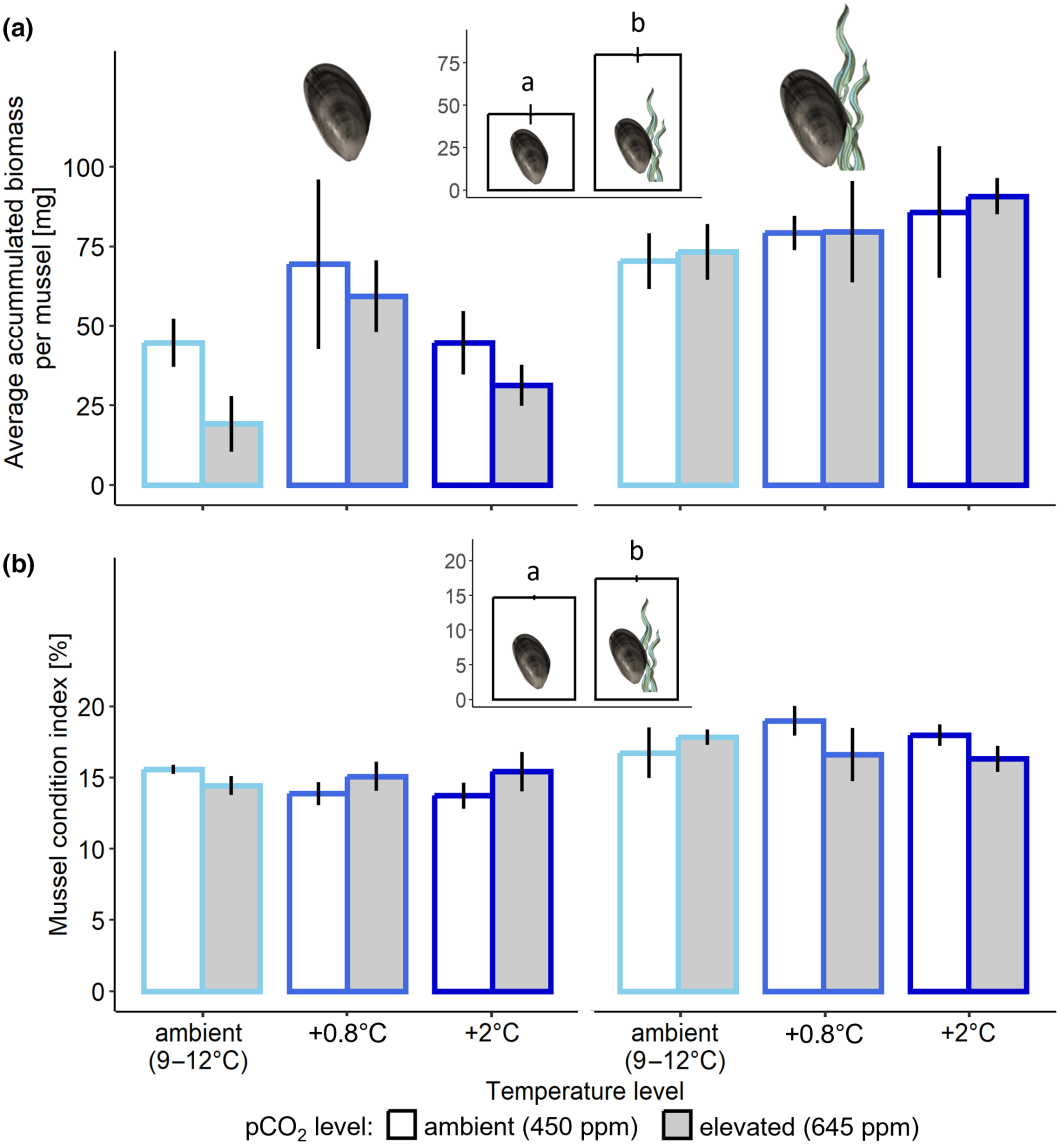
Mean (±SE) of accumulated biomass per mussel (a) and condition index of mussels (b), at winter ambient (light blue), +0.8°C (blue) and +2°C (dark blue) temperature, and at ambient (white bars) and elevated (grey bars) pco_2_, in the absence (left panel) or presence (right panel) of algae, *n* = 5. Insets show significant differences amongst groups of means without and with algae, also indicated by lowercase letters (*p* < .001).

Temperature had no effect on mussel biomass (*F*_2,48_ = 2.398; *p* = .102; Figure 4a), nor on the condition index (*F*_2,48_ = 0.086; *p* = .917; Figure 4b), nor did pco_2_ (*F*_1,48_ = 0.821, *p* = .369 and *F*_1,48_ = 0.093; *p* = .862; Figure 4). The presence of algae, however, had a positive effect on mussel biomass (*F*_1,48_ = 22.073; *p* < .001; Figure 4a) and condition index (*F*_1,48_ = 18.437; *p* < .001; Figure 4b). Mussel biomass almost doubled in the presence of algae compared to treatments without algae (Figure 4a) and the mussel condition index was almost 20% greater in the presence of algae compared to their absence (Figure 4b).

**FIGURE 5:**
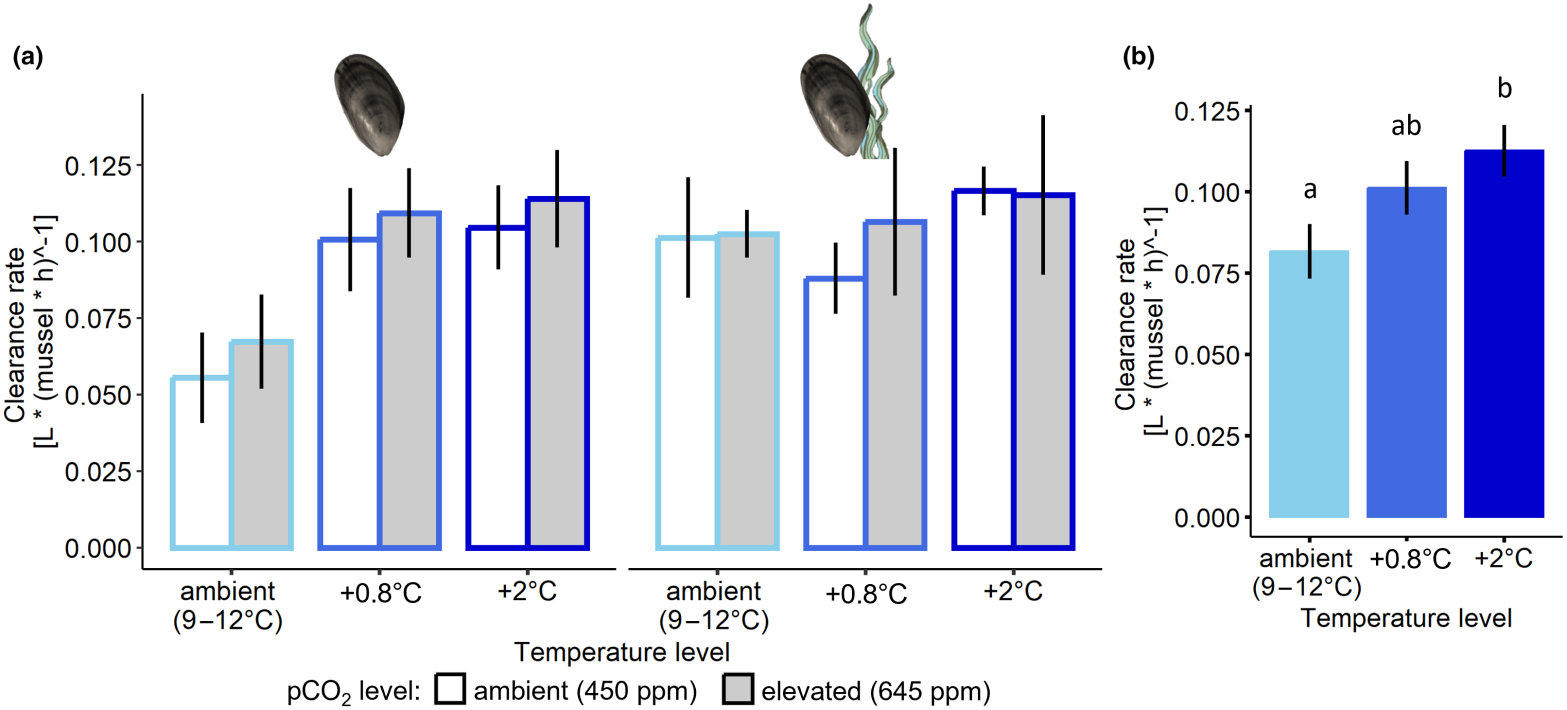
(a) Mean (±SE) mussel clearance rates after 17 h (all treatments) at winter ambient (light blue), +0.8°C (blue) and +2°C (dark blue) temperature, and at ambient (white bars) and elevated (grey bars) pco_2_, in the absence (left panel) or presence (right panel) of algae, *n* = 5 and (b) shows the effect of temperature on the mean (±SE) of mussel clearance rates based on these pooled treatments where groups of means that are significantly different are indicated by lowercase letters (*p* < .05).

No differences amongst treatments were found in clearance rates after 2 h of feeding (temperature: *F*_2,48_ = 2.213, *p* = .120; pco_2_: *F*_1,48_ = 3.156, *p* = .082; algae: *F*_1,48_ = 0.750, *p* = .391), however, after 17 h, a significant effect of temperature on mussel clearance rates (Figure 5a) was identified (*F*_2,48_ = 3.556; *p* = .036), with an almost 40% greater rate in the warmest temperature level compared to the ambient temperature level (Figure 5b). No effects of pco_2_ (*F*_1,48_ = 0.708; *p* = .404) nor the presence of algae (*F*_1,48_ = 1.870; *p* = .178) were identified after 17 h.

No effects of temperature, pco_2_ nor the presence/absence of algae on mussel shell strength (temperature: *F*_2,43_ = 0.454, *p* = .638; pco_2_: *F*_1,43_ = 0.000, *p* = .995; algae: *F*_1,43_ = 0.774, *p* = .384) or byssus strength (temperature: *F*_2,22_ = 0.058, *p* = .944; pco_2_: *F*_1,22_ = 0.685, *p* = .417; algae: *F*_1,22_ = 0.201, *p* = .658) were found.

### 3.2 Algal responses

**FIGURE 6:**
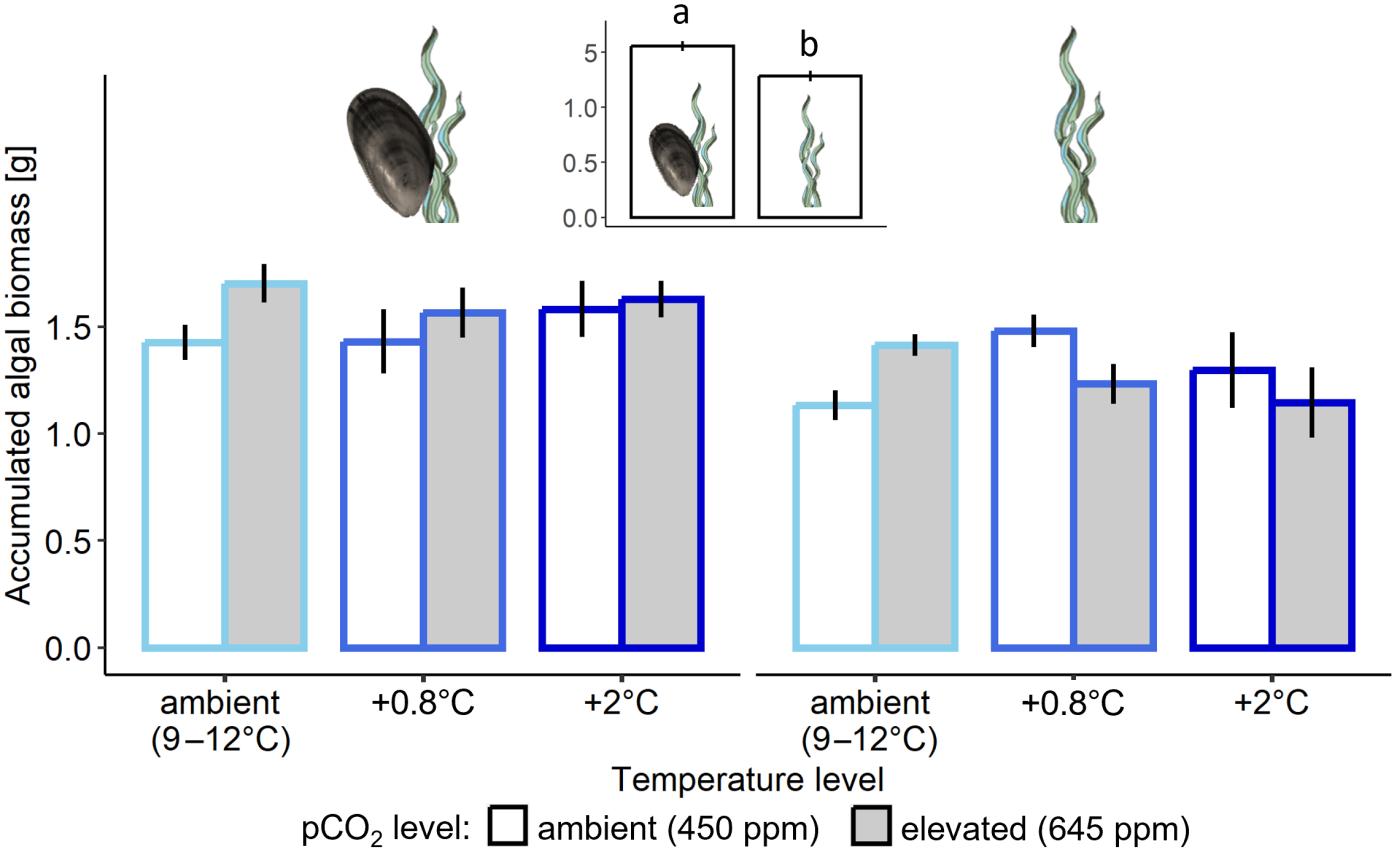
Mean (±SE) accumulated biomass of algae at winter ambient (light blue), +0.8°C (blue) and +2°C (dark blue) temperature, and at ambient (white bars) and elevated (grey bars) pco_2_, in the presence (left panel) or absence (right panel) of mussels, *n* = 5 per bar. Inset shows significant differences amongst groups of means with and without mussels, also indicated by lowercase letters (*p* < .001).

Most of the algal response variables quantified were not affected by changes in temperature or pco_2_ and no interactive effects were identified amongst any of the factors on any of the algal response variables. Specifically, neither temperature (*F*_2,48_ = 0.014; *p* = .986) nor pco_2_ (*F*_1,48_ = 0.734; *p* = .396) affected the accumulated biomass of algae in the mesocosms (Figure 6). In the presence of mussels, however, 20% more algal biomass accumulated than in the absence of mussels (*F*_1,48_ = 17.156; *p* < .001; Figure 6).

At the end of the experiment, no effects of temperature (*F*_2,29_ = 5.161; *p* = .527) nor pco_2_ (*F*_1,29_ = 0.000; *p* = 1.000) on the abundance of kelp individuals were identified, however, sporophytes were only visible in the treatments without mussels. It is unclear if no sporophytes survived or if they remained too small for identification under a microscope in the treatments with mussels.

**FIGURE 7:**
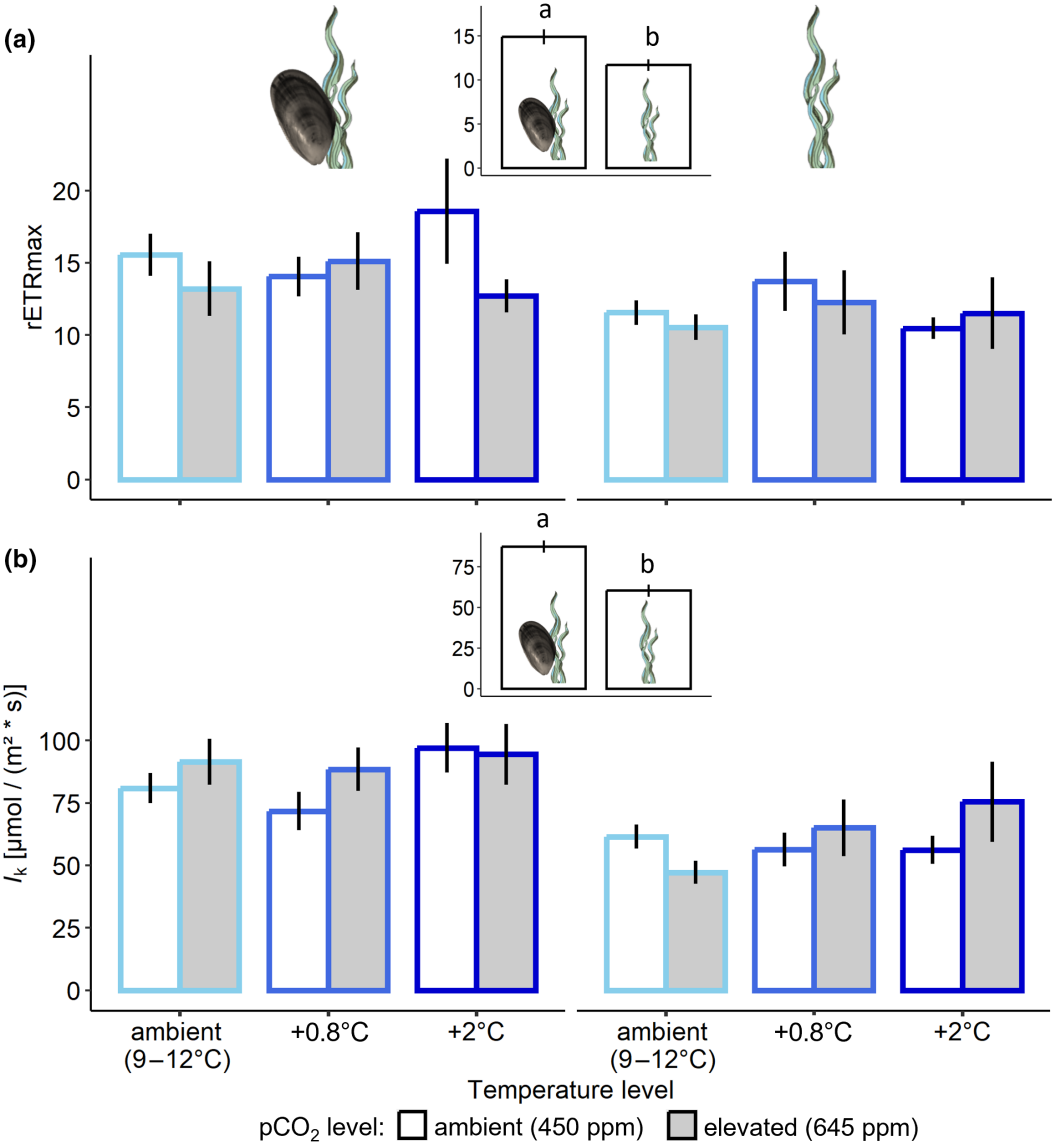
Mean (±SE) relative maximum electron transport rate (a) and saturating light intensity (b), at winter ambient (light blue), +0.8°C (blue) and +2°C (dark blue) temperature, and at ambient (white bars) and elevated (grey bars) pco_2_, in the presence (left panel) or absence (right panel) of mussels; *n* = 5. Insets show significant differences amongst groups of means with and without mussels, also indicated by lowercase letters (*p* < .001).

Results of the rapid light curves revealed no effects of temperature nor pco_2_ on the relative maximum electron transport rate rETRmax (temperature: *F*_2,48_ = 0.324; *p* = .725; pco_2_: *F*_1,48_ = 2.130; *p* = .151; Figure 7a) or on the saturating light intensity *I*_k_ (temperature: *F*_2,48_ = 1.459; *p* = .243; pco_2_: *F*_1,48_ = 0.774; *p* = .383; Figure 7b) of algae in these treatments (Figure 6). The presence of mussels, however, significantly affected rETRmax (*F*_1,48_ = 10.107; *p* = .003; Figure 7a) and *I*_k_ (*F*_1,48_ = 31.966; *p* < .001; Figure 7b). rETRmax increased by 30% in the presence of mussels and *I*_k_ by 45%. The light harvesting efficiency *α* did not show any differences amongst experimental treatments (temperature: *F*_2,48_ = 1.400, *p* = .257; pco_2_: *F*_1,48_ = 2.790, *p* = .101; mussels: *F*_1,48_ = 3.234, *p* = .078).

**FIGURE 8:**
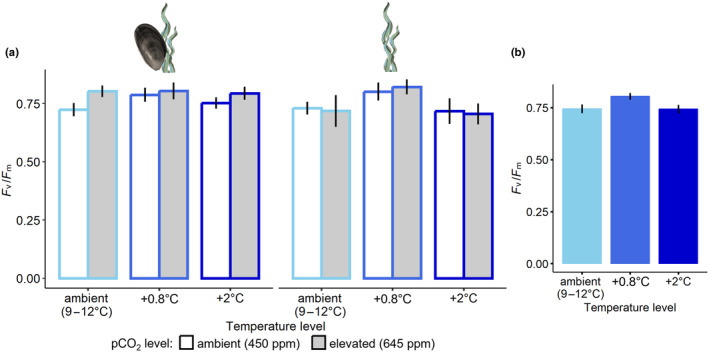
(a) Mean (±SD) maximum quantum yield of algae (all treatments) at winter ambient (light blue), +0.8°C (blue) and +2°C (dark blue) temperature and at ambient (white bars) and elevated (grey bars) pco_2_, in the presence (left panel) or absence (right panel) of mussels, *n* = 5 and (b) shows the significant effect of temperature on the maximum quantum yield of algae based on pooled treatments.

The maximum quantum yield *F*_v_/*F*_m_ (Figure 8a) was significantly affected by temperature (*F*_2,48_ = 3.261; *p* = .047; Figure 8b), but not by pco_2_ (*F*_1,48_ = 0.972; *p* = .329) nor the presence or absence of mussels (*F*_1,48_ = 1.602; *p* = .212). Post‐hoc tests suggest that the medium temperature level tended towards a significant difference from the ambient (*p* = .085) and the warmest level (*p* = .074; Table A.2.1: Appendix S1).

### 3.3 Total accumulated biomass (of mussels and algae)

**FIGURE 9:**
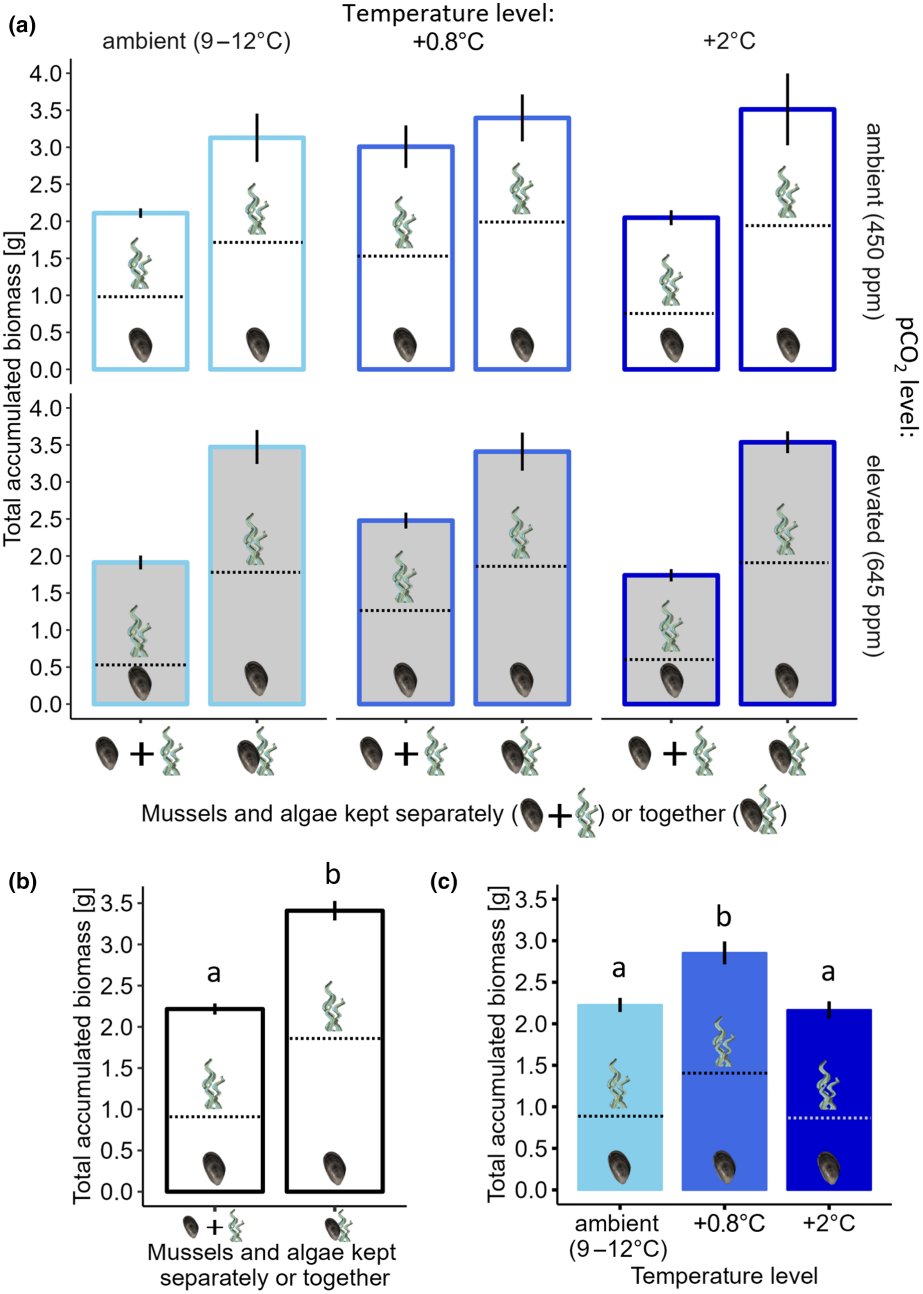
(a) Mean (±SE) total accumulated biomass per mesocosm in all treatments, based on the sum of mussel and algal biomass kept in separate treatments (*n* = 25) compared to the total accumulated biomass of mussels and algae kept together (*n* = 5). The proportional contributions of mussels or algae to the total combined biomass accumulated are indicated by dashed lines. The panels show means at winter ambient (light blue), +0.8°C (blue) and +2°C (dark blue) temperature, the rows at ambient (white bars) and elevated (grey bars) pco_2_; (b) Shows the significant effects of functional group (mussels and algae kept separately vs. together) on total combined biomass and (c) shows the effects of temperature on total combined accumulated biomass. Significant differences amongst groups of means are indicated by lowercase letters (*p* < .05).

Temperature affected the total accumulated biomass of mussels and algae (*F*_2,228_ = 16.314; *p* < .001; Figure 9a) and post‐hoc tests indicated significant differences between the ambient and the medium (+0.8°C; *p* = .003) and between the medium and the warmest (+2°C; p < .001) temperature level but not between ambient and warmest, thus, total combined biomass peaked in the medium temperature treatment (Figure 9c). pco_2_ had no effect on the total accumulated biomass (*F*_1,176_ = 2.449; *p* = .118). None of the abiotic factors manipulated interacted when comparing the total accumulated biomass of mussels and algae in treatments where they were kept together to the sums of biomass in treatments that contained just mussels or algae. Whether mussels and algae were kept separately or together affected their total accumulated biomass significantly (*F*_3,228_ = 135.902; *p* < .001), which was 54% greater when kept together compared to their sums when kept separately. Differences in total accumulated combined biomass were mainly driven by mussels (Figures 6 and 9b).

An overview of all statistical results is presented in Table 1.

**TABLE 1:** Statistical results of the effects of warming and pco_2_ on mussels and algae kept separately or together.

Response variable	Factor interactions	Temperature effect		pco_2_ effect		Effect of taxa present	
		*F*	*p*	*F*	*p*	*F*	*p*
Mussel mortality	–	*F*_2,48_ = 6.382	**.004**	*F*_1,48_ = 0.37	0.549	*F*_1,48_ = 8.403	**.006**
		Significantly higher (*p* = .002) at +2°C than at winter ambient				Significantly higher in the absence of algae	
Mussel biomass	–	*F*_2,48_ = 2.398	.102	*F*_1,48_ = 0.821	.369	*F*_1,48_ = 22.073	**<.001**
						Significantly higher in the presence of algae	
Mussel condition index	–	*F*_2,48_ = 0.086	.917	*F*_1,48_ = 0.093	.862	*F*_1,48_ = 18.437	**<.001**
						Significantly higher in the presence of algae	
Mussel clearance rates after 17 h	–	*F*_2,48_ = 3.556	**.036**	*F*_1,48_ = 0.708	.404	*F*_1,48_ = 1.870	.178
		Significantly higher (*p* = .030) at +2°C than at winter ambient					
Mussel shell strength	–	*F*_2,43_ = 0.454	.638	*F*_1,43_ = 0.000	.995	*F*_1,43_ = 0.774	.384
Mussel byssus strength	–	*F*_2,22_ = 0.058	.944	*F*_1,22_ = 0.685	.417	*F*_1,22_ = 0.201	.658
Algal biomass	–	*F*_2,48_ = 0.014	.986	*F*_1,48_ = 0.734	.396	*F*_1,48_ = 17.156	**<.001**
						Significantly higher in the presence of mussels	
Kelp abundance	–	*F*_2,29_ = 5.161	.527	*F*_1,29_ = 0.000	1.000	Only visible without mussels	
Algal rETRmax	–	*F*_2,48_ = 0.324	.725	*F*_1,48_ = 2.130	.151	*F*_1,48_ = 10.107	**.003**
						Significantly higher in the presence of mussels	
Algal *I*_k_	–	*F*_2,48_ = 1.459	.243	*F*_1,48_ = 0.774	.383	*F*_1,48_ = 31.966	**<.001**
						Significantly higher in the presence of mussels	
Algal *α*	–	*F*_2,48_ = 1.400	.257	*F*_1,48_ = 2.790	.101	*F*_1,48_ = 3.234	.078
Algal *F*_v_/*F*_m_	–	*F*_2,48_ = 3.261	**.047**	*F*_1,48_ = 0.972	.329	*F*_1,48_ = 1.602	.212
		Higher at +0.8°C than at ambient (*p* = .085) and +2°C (*p* = .074)					
Total biomass	–	*F*_2,228_ = 16.314	**<.001**	*F*_1,176_ = 2.449	.118	*F*_3,228_ = 135.902	**.001**
		Significantly higher at +0.8°C than at ambient (*p* = .003) and +2°C (*p* < .001)				Significantly higher when kept together	

*Note*: Significant *p* values are highlighted in bold.

## 4 DISCUSSION

Understanding how species interact when exposed to multiple stressors associated with climate change is crucial to estimating future ecosystem structure and the provisioning of ecosystem services. Here, we show that moderate predicted warming in winter, but not acidification, affected the functioning of mussels and algae and that culturing them together enhanced both their performances compared to treatments that contained only mussels or only algae.

Mussel clearance rates were greater at warmer temperatures as we hypothesised. Contrastingly, mussel mortality was also greater, which was contrary to our expectations because the warmed winter temperatures were within the seasonally experienced temperature range of the mussels. Recent winter mass mortality events, for which no putative causes could be identified, highlight the importance of investigating the effect of changing winter conditions on coastal biodiversity (Capelle et al., 2021; Polsenaere et al., 2017). Increased mortality at warmer winter temperatures indicates that the mussels in our experiment were not able to maintain their fitness despite increasing their energy intake. The average accumulated biomass of individual mussels and condition index were not affected by temperature. The fact that marine ectotherms, including bivalves and algae, adapt their metabolic rates and directly related traits, such as food clearance rates in the case of mussels, according to the prevailing water temperature within their thermal range is well understood (e.g. Gao et al., 2019; Kittner & Riisgård, 2005; Pörtner, 2010; Roma et al., 2021). When the food supply is high, biomass in *M. edulis* usually increases accordingly, whilst the condition index can decrease in response to warming when food is limited (Mackenzie et al., 2014; Thomsen et al., 2013). The condition index generally compares recent physiological activity (mussel flesh weight) to longer‐term, cumulative growth (mussel shell weight) and is a proxy for the net response following changes in food consumption rate, respiration rate, growth rate, biomass turnover rate, calcification rate and so on (Lucas & Beninger, 1985). The lower the index, the higher the recently experienced stress. In our experiment, mussel deaths were recorded throughout the experiment, whilst biomass and condition index were only recorded in individuals that were alive at the end. The dosage of food supplied remained the same across all treatments. When mussels died during our experiment, the relative availability of food increased for the remaining mussels, that is, surviving individuals at higher temperatures had access to larger food shares compared to individuals in treatments at lower temperatures where the same food dosage had to cover the requirements of more surviving individuals. We suspect that this relatively higher food availability may have served to compensate for increased energy demands at higher temperatures, for example, required for byssus production, and may explain the absence of temperature effects on individual biomass and condition index (Roberts & Carrington, 2023).

The absence of pco_2_ effects on mussel responses may be because coastal ecosystems experience large daily and seasonal fluctuations in pco_2_ and respective pH conditions (Duarte et al., 2013; Fernández et al., 2019; Lutier et al., 2022; Vargas et al., 2017). When the mussel spat used in our experiment was collected in October 2020, a snapshot measurement of in‐situ pH of 8.24 was taken, that is, 0.15 logarithmic units higher than in the ambient treatment in the presented experiment, which is a greater interval than between the ambient and elevated pco_2_ level applied. Long‐term, continuous seasonal monitoring data of the pH of Killary Fjord is not available. Between 2007 and 2009, however, summer pH ranged from 7.87 to 8.30 (O'Boyle et al., 2013), reflecting the influences of groundwater from surrounding calcareous limestone, nutrient inputs and biological activity (Duarte et al., 2013; McGrath et al., 2016). Therefore, the experimental pco_2_ level of 645 ppm with an associated decrease in pH of 0.1 units compared to the ambient level of 450 ppm, or pH 8.09, respectively, lies within the range that the mussels experience and are likely adapted to in their natural habitats (Melzner et al., 2013; O'Boyle et al., 2013; Thomsen et al., 2013). It is currently unknown if natural carbon chemistry fluctuations will simply shift according to future mean background acidification, or if fluctuations will become more extreme. The effects of both possibilities should be included in future acidification research, given that increased temperature variation may be more harmful to ectotherms than increased mean temperature (Pansch & Hiebenthal, 2019; Vasseur et al., 2014). Furthermore, biological processes, such as growth and calcification, in juvenile *M. edulis* are mainly driven by food abundance and are not impacted by pco_2_ levels of up to 3350 ppm when the food supply is high (Melzner et al., 2011; Thomsen et al., 2013). Similar to our results, increased clearance rates were found in individual juvenile *M. chilensis* at +4°C of warming, whereas acidified conditions of 700 ppm did not have any effects compared to ambient (380 ppm) conditions (only highly acidified conditions at 1000 ppm reduced the clearance rates independent of temperature; Navarro et al., 2016).

We found no effect of any of the experimental treatments on shell strength. Other studies found evidence of shell dissolution in morphometric analyses and/or weakened shell strength in crushing tests after 6–9 months of exposure to strong acidification (>2400 ppm or >−0.4 pH units) when mussels were kept at poor food supply (Mackenzie et al., 2014; Melzner et al., 2011). Similarly, oysters grown in acidified conditions grew slower than control individuals, which made them more susceptible to predation (Sanford et al., 2014). Our results show that a 1‐year‐old spat can resist shell degradation under moderate ocean acidification for at least 6 weeks when kept at a favourable food supply.

Although byssal thread production was not quantified formally as part of our study, there was no evidence to suggest that it was affected by any of the experimental treatments. Previous studies suggested that byssus strength is highly size‐dependent and that the attachment strength of juvenile life stages may be less affected by ocean acidification than that of larger adults (Clements & George, 2022). The absence of any effects of all applied treatments aligns with other recent conclusions that byssus production is generally prioritised over other energy expenditure, such as growth, in mussels in stressful conditions (Roberts & Carrington, 2023).

Similar to the pattern found in mussels, algal energy uptake as *F*_v_/*F*_m_ depended on temperature but not pco_2_, and there was no effect of temperature on accumulated algal biomass. This may indicate that either the maximum quantum yield potential was not fully exploited, or that another cellular maintenance was prioritised over biomass and growth despite favourable temperatures. It is unclear if co‐limitation of another resource (e.g. light) impaired algal growth under elevated pco_2_ (Connell et al., 2013). According to Young, Doall, and Gobler (2022), *S. latissima* may benefit double from pco_2_ elevated ≥830 ppm through enhanced growth and reduced herbivory, which was beyond our experimental design to test.

The total accumulated biomass of mussels and algae combined was significantly greater at +0.8°C than at winter ambient or the warmest (+2°C) temperature. The proportional contributions of mussels to total accumulated biomass and the absence of a temperature effect on accumulated algal biomass suggest that differences in total accumulated biomass were driven by the increase in mussels. Mussel clearance rates and mortality were significantly greater at +2°C than at winter ambient temperature, indicating an increase in temperature. At the medium temperature level of +0.8°C warming, mussels apparently still managed to fulfil their increased energy demand and, therefore, died less often than at the highest temperature level of +2°C warming, resulting in the greatest total accumulated biomass. Whilst total accumulated biomass at +2°C resembled that of winter ambient temperature, the underlying mussel population contained fewer individuals. This shows that small intervals of warming between 0.8 and 2°C, even at the lower end of seasonally experienced temperatures, can have significant effects on community composition, which may have profound ecological consequences.

A key finding of this study was that the presence of algae enhanced mussel performance by decreasing mussel mortality and increasing mussel biomass and condition index. Concurrently, the presence of mussels also enhanced algal performance by increasing algal biomass and strengthening photosynthetic adaptation (relative maximum electron transport rate rETRmax and the saturating light intensity *I*_k_). This mutual facilitation is exemplified in particular by comparing the total accumulated biomass in treatments where both mussels and algae were present to the sums of biomass in treatments comprised of only mussels and only algae. Here, we have identified a synergistic interaction, where the total biomass of mussels and algae grown together exceeded the sums of biomass from treatments in which only mussels or only algae were grown by up to 54%, which could be considered a form of complementarity or transgressive overyielding (Schmid et al., 2008). We know that benthic diatoms dominate the biofilms that produce extracellular polymeric substances binding particles together, which play an important role as a resuspended food source for filter feeders including mussels (Andriana et al., 2021; Evrard et al., 2012; Kang et al., 2006). In turn, the mussels enrich the biofilms with nutrients by depositing ammonium, faeces and pseudofaeces, which fuels benthic primary production and diatom growth, and eventually benefits mussels, for example, by increasing sediment surface stability and sedimentation (Andriana et al., 2021; Lindström Swanberg, 1991). In the present study, it remains unclear if mussel mortality decreased in the presence of algae because of additional food available through re‐suspended microphytobenthos, or by an improved environment for decomposing microorganisms that metabolised waste products, such as ammonia, and, therefore, increased water quality. The microalgae in the treatments with mussels and algae were fed both with f2 medium, mussel faeces and pseudofaeces, which led to increased algal biomass accumulation compared to treatments in which mussels were absent. Simultaneously, however, the microalgae in these treatments seem to have outcompeted the juvenile kelp sporophytes, presumably owing to their ability for faster growth. Kelp exposed to various environmental stressors including warming, acidification and predation tends to be outcompeted by turf algae, which has repeatedly caused the change from previously kelp‐dominated ecosystems to then degraded habitats dominated by turfy algal mats as alternate ecological states (Connell et al., 2013; Connell & Russell, 2010). Identification of the mechanisms underpinning this increase in algal biomass in the presence of mussels, that is, whether it is direct facilitation or indirectly by altering associated biofilm, requires further investigation. Rossoll et al. (2012) showed that copepod growth and reproduction changed according to algal fatty acid concentration and composition that was affected by pco_2_ levels. Our results of algal photo physiology align with Rugiu et al. (2020) who exposed 1‐year‐old *S. latissima* to mussel farm effluent and found increased rETRmax and *I*_k_ (but no effect on the light harvesting efficiency *α*) compared to control treatments. Having found the highest biomass of both mussels and algae in treatments where they were grown together compared to treatments with only mussels or only algae, our study contributes to the evidence that co‐cultivation of species as practiced in integrated multi‐trophic aquaculture may be synergistically beneficial and enhance overall yield (Hargrave et al., 2022).

## 5 CONCLUSION

Our study showed that short‐term metabolic processes related to energy intake (mussel clearance rates or algal maximum quantum yield) increased with moderately elevated temperature, whilst moderately elevated pco_2_ did not have any effect on the response variables. Simultaneously, mussel mortality increased with winter warming, resulting in the greatest total accumulated biomass (of mussels and algae combined) at the medium temperature level of ambient +0.8°C. We show that, depending on species' positions on their thermal performance curves and in their thermal ranges, future marine communities in a warmed ocean are likely to undergo severe changes in their structure and functioning, even if changes in temperature are 0.8–2°C and may seem small. The tolerance of mussels towards elevated pco_2_ and lowered pH, when food is abundant, highlights the importance of considering ecosystem dynamics and trophic interactions under global change. Furthermore, considering that 645 ppm CO_2_ did not impact the performance of juvenile mussels negatively, and that marine organisms will be exposed to more frequent and more intense environmental fluctuation extremes, there is potential for cross‐generational adaptation to more acidic conditions in the future. Whilst cross‐generational adaptation was not tested in this study, it should be investigated in future studies, possibly including transplantations of broodstock reared under different conditions with gradual stressor introduction (Byrne et al., 2020).

Efforts to include facilitation into theory, models and paradigms of population and community ecology are ongoing (Bruno et al., 2003) and have been further emphasised by recent calls to include facilitative interactions in mediating climate change impacts on biodiversity (Kéfi et al., 2024). Our empirical results contribute directly to these endeavours. We show that mussels and algae mutually facilitated their performance, including overall productivity and energy budgets (mussel condition index, both mussel and algal biomass, algal electron transport rate and light sensitivity). Considering that the total biomass of mussels and algae grown together substantially exceeded the biomass of sums of treatments with only mussels and only algae present, and that mussel mortality was significantly reduced where algae were present, efforts to increase and conserve biodiversity in marine ecosystems may provide noticeable ecological and economic benefits.

## AUTHOR CONTRIBUTIONS

**Katrin S. H. Schertenleib:** Conceptualization (lead); formal analysis (lead); investigation (lead); methodology (lead); visualization (lead); writing – original draft (lead); writing – review and editing (equal). **Tallulah Davey:** Investigation (supporting); methodology (supporting); writing – review and editing (supporting). **David Taylor:** Investigation (supporting); methodology (supporting); writing – review and editing (supporting). **Nessa E. O'Connor:** Conceptualization (supporting); methodology (supporting); writing – review and editing (equal).

## Supporting information


Appendix S1.

## Data Availability

The data that support the findings of this study are openly available in “Data Dryad” at https://doi.org/10.5061/dryad.vdncjsz3b. The R code used to process the data is available at https://github.com/katmarsci/MesocosmExperiment‐Mussels‐Algae_2020‐10to12_pub/releases/tag/v1.0.0_datalink.
