# Supplementary material for: Key benthic species are affected by predicted warming in winter but show resistance to ocean acidification
Source: Ecol Evol. 2024 Sep 18;14(9):e70308. doi: 10.1002/ece3.70308 (PMC11410397; doi:10.1002/ece3.70308)
Supplement: Supplementary file 1 — Appendix S1. [file ECE3-14-e70308-s001.docx]

# Appendix – Supporting Information

Corresponding article: Katrin S. H. Schertenleib, Tallulah Davey, David Taylor, Nessa E. O’Connor. 2024. The role of mutualism in marine benthic communities: Key species are affected by predicted warming but show resistance to ocean acidification. Ecology and Evolution

## Shell length determination and size-independent mortality

At the start and the end of the experiment, the mussels from each mesocosm were photographed on graph paper for subsequent shell length determination. Initial and final individual mussel shell lengths were determined from a random half of the mesocosms (*n* = 30) by measuring the maximum anterior-posterior axes (Seed, 1968) using image analysis (ImageJ 1.53q; Rasband, 1997). Mussel lengths were comparable within photos but not across photos because the camera was readjusted several times when photos were taken. As a consequence, no absolute length growth rates could be determined. However, Kolmogorov Smirnov tests confirmed that in 29 of the 30 examined mesocosms the lengths distributions remained the same throughout the experiment, i.e. mussel mortality was independent of size (Table A.2.2). Accordingly, mussel individuals of a mesocosm were treated as similar on average and clearance rate samples that were taken before mussel retrieval at the end of the experiment were standardised by the number of alive mussels per mesocosm.

Shell lengths of mussels used in shell strength tests were determined separately with the same methodology and without camera readjustments to ensure comparability of the lengths.

References A.1:

Rasband, W. S. (1997). ImageJ. U. S. National Institutes of Health, Bethesda, Maryland, USA. Retrieved from https://imagej.nih.gov/ij/

Seed, R. (1969). The ecology of Mytilus edulis L. (Lamellibranchiata) on exposed rocky shores - I. Breeding and settlement. Oecologia, 3(3–4), 277–316. https://doi.org/https://doi.org/10.1007/BF00390380

## Supplemental Tables

Table A.2.1 TukeyHSD test on the effect of temperature (3 levels: Ta – ambient, T+ – ambient +0.8 °C, T++ – ambient + 2°C) on the maximum quantum yield F_v_/F_m_.

|  | Diff | Lower 95% CI | Upper 95% CI | *P (adj)* |
| --- | --- | --- | --- | --- |
| T++-T+ | -0.062 | -0.128 | 0.005 | 0.074 |
| Ta-T+ | -0.060 | -0.126 | 0.007 | 0.085 |
| Ta-T++ | 0.002 | -0.065 | 0.068 | 0.998 |

Table A.2.2 Two-sample Kolmogorov-Smirnov tests comparing the initial and final mussel length distributions in 30 of 60 mesocosms

| Mesocosm ID | D | *P* |
| --- | --- | --- |
| B1 | 0.200 | 0.723 |
| B6 | 0.217 | 0.559 |
| B9 | 0.179 | 0.812 |
| C1 | 0.400 | 0.048 |
| C4 | 0.110 | 0.998 |
| C5 | 0.140 | 0.952 |
| E2 | 0.133 | 0.962 |
| E6 | 0.125 | 0.970 |
| E9 | 0.173 | 0.807 |
| F3 | 0.153 | 0.906 |
| F4 | 0.242 | 0.358 |
| F6 | 0.110 | 0.998 |
| G1 | 0.122 | 0.984 |
| G8 | 0.162 | 0.964 |
| G9 | 0.230 | 0.571 |
| H1 | 0.163 | 0.736 |
| H3 | 0.259 | 0.249 |
| H7 | 0.138 | 0.989 |
| H8 | 0.186 | 0.761 |
| I1 | 0.130 | 0.957 |
| I4 | 0.212 | 0.585 |
| I5 | 0.236 | 0.600 |
| J2 | 0.167 | 0.893 |
| J4 | 0.264 | 0.453 |
| J5 | 0.125 | 0.980 |
| J7 | 0.137 | 0.973 |
| J9 | 0.176 | 0.862 |
| K3 | 0.250 | 0.441 |
| K5 | 0.208 | 0.436 |
| K9 | 0.208 | 0.637 |
